# Barriers to Voice Behavior in Online and Hybrid Multidisciplinary Team Meetings: A Qualitative Study

**DOI:** 10.1111/jep.70614

**Published:** 2026-10-11

**Authors:** Karin Kee, Stephanie M. E. van der Burgt, Marike L. D. Broekman, Rob J. A. Nabuurs

**Affiliations:** ^1^ Department of Organization Sciences Vrije Universiteit Amsterdam the Netherlands; ^2^ Teaching and Learning Center (TLC), Amsterdam University Medical Center University of Amsterdam Amsterdam the Netherlands; ^3^ Amsterdam Public Health Section Quality of Care Amsterdam the Netherlands; ^4^ Department of Neurosurgery Haaglanden Medical Center The Hague the Netherlands; ^5^ Department of Neurosurgery Leiden University Medical Center Leiden the Netherlands

## Abstract

**Rationale:**

Guidelines for the treatment of a variety of diseases and medical conditions increasingly call for decision‐making by a multidisciplinary team, which typically takes place during multidisciplinary team meetings (MDTMs). However, studies examining in‐person MDTMs show that the decision‐making processes during such meetings often fall short of optimal performance due to participants refraining from exercising voice behavior—denoting the communicative act of sharing one's perspectives, suggestions, and concerns related to the patient's diagnosis and treatment options with fellow participants. The expanding digitalization and the COVID‐19 pandemic have led to an increase in online and hybrid meetings, potentially introducing new obstacles to participants' voice behavior.

**Aims and Objectives:**

The present paper aims to explore the challenges encountered by participants of online and hybrid MDTMs in exercising their voice behavior.

**Methods:**

Observations of neuro‐oncology MDTMs in two Dutch hospitals and interviews with chairpersons and participants of the observed MDTMs.

**Results:**

Respondents encountered difficulties in expressing their perspectives, ideas, and concerns during online and hybrid meetings, which could be attributed to (1) the absence of non‐verbal cues, (2) technological deficiencies, (3) a personal lack of focus, (4) suboptimal layout of the meeting room, and (5) challenges in building relationships with other attendees.

**Conclusions:**

Obstacles to voice behavior in MDTMs are multifaceted, with specific barriers inherent to online and hybrid meetings that are not present in in‐person meetings.

## 1 Introduction

Multidisciplinary team meetings (MDTMs) have emerged as an indispensable component of contemporary healthcare [1, 2]. During MDTMs, a diverse team of medical experts convenes to discuss and determine the diagnosis and/or treatment plan for individual patients with cancer and other complex diseases [3, 4]. By harnessing the collective knowledge, expertise, and experience of various healthcare professionals, MDTMs hold promise for improving diagnostic accuracy and thoroughness and for identifying an optimal treatment plan tailored to a patient's condition, needs, and preferences [4, 5]. Against this backdrop, national guidelines increasingly require patients to be discussed during MDTMs [6, 7].

However, studies strongly indicate that decision‐making processes during MDTMs often fall short of optimal performance. Extensive social psychological research shows that effective decision‐making, regardless of the setting, requires opportunities and the willingness for thorough discussion, the development of shared understanding, and the integration of diverse perspectives among participants [8]. These processes depend fundamentally on effective *voice behavior*, defined as sharing one's perspectives, suggestions, and concerns regarding patient diagnosis and treatment options with other members [9]. When MDTM members remain silent, they cannot contribute their expertise or challenge existing views, leading to fragmented decision‐making and hindering the achievement of high‐quality, evidence‐based, patient‐centered decisions [10, 11, 12].

Unsurprisingly, in recent years, the topic of *voice behavior* has received growing attention from MDTM scholars. Studies in this regard show that participants, particularly those lower in status and hierarchy (e.g., nurses) may fear negative repercussions or anticipate that their perspectives will not be listened to or given due consideration [11, 12]. Exemplifying this, Horlait et al. [4] found that MDTMs are typically led by medical specialists, with minimal input from nurses and other non‐medical staff, resulting in psychosocial factors and patients' individual preferences and needs remaining unaddressed. Similarly, with the rapid increase in the number of patients to be discussed, attributed to the rising incidence and prevalence of cancer [7], time pressure increases. When MDTM participants experience time constraints, their inclination to voice decreases because they believe that doing so may take too much time [9, 13].

While researchers have begun to explore obstacles to participants' voice behavior during in‐person MDTMs [10, 11, 12], the ongoing wave of digitalization and the COVID‐19 pandemic have prompted a significant shift towards online and hybrid meeting formats using videoconferencing software [14]. Given that research indicates that the context in which decision‐making occurs can influence actors' voice behavior [9], the shift to online/hybrid MDTMs may have introduced novel challenges that impede participants' ability or willingness to voice their perspectives, which they did not encounter in in‐person meetings. Understanding participants' experiences with exercising voice in online and hybrid MDTMs, with a focus on the barriers they encounter, is vital for safeguarding the effectiveness and overall quality of decision‐making in these meetings. Therefore, in the present paper, we explore the following question: *“What barriers do participants in online and hybrid MDTMs experience when trying to exercise voice behavior?”*

## 2 Methods

### 2.1 Design

We conducted a qualitative study using observations and interviews to answer our research question. This design was deemed suitable because observations enabled researchers to obtain firsthand accounts of decision‐making processes in online and hybrid settings, while interviews provided an avenue to delve deeper into participants' personal experiences. Combining observations and interviews further allowed for data triangulation, enhancing the reliability and validity of the findings by cross‐verifying information obtained through different methods [15].

The study reported here is part of a larger study examining decision‐making processes during MDTMs.

### 2.2 Setting

The study involved the neuro‐oncology care teams of two of the thirteen hospitals in the Netherlands that specialize in neuro‐oncological surgery. In Hospital A, a university medical center, MDTMs were conducted twice weekly. The MDTMs were fully online, meaning that all participants were seated at their own computers, either at their workplaces or at home. The MDTMs in Hospital A typically lasted 45 min, during which 8 to 12 patients were discussed. Hospital B, a leading clinical hospital, adopted a hybrid model in which care professionals worked across two locations. At both locations, participants gathered in dedicated meeting rooms and used a videoconferencing platform for communication. MDTMs at Hospital B were held weekly and generally lasted 1 h, during which 15–20 patients were discussed.

In both hospitals, MDTMs were attended by at least one neurosurgeon, neurologist, radiation and medical oncologist, neuroradiologist, neuropathologist, and specialized nurse. Junior doctors were also present since MDTMs offer an ideal learning opportunity [16]. In both hospitals, a rotating chairmanship system was used, with two neurosurgeons and two neurologists at Hospital A and two neuro‐oncologists at Hospital B, respectively, serving as chairpersons.

### 2.3 Data Collection

Data collection took place in spring 2022 and was conducted by the first author, with assistance from three research assistants who had received training in conducting observations and interviews.

In both hospitals, data collection commenced with non‐participant observations, in which the researchers assumed an observational role without intervening in the MDTMs [17]. While observing, the researchers paid attention to several things, including participant engagement (i.e., attentiveness), communication patterns (i.e., who participated in discussions), the influence of technology (i.e., how the videoconferencing systems were used, possible technical difficulties), and the chairperson's role (i.e., how the chairs guided the discussion). After each MDTM, the researchers wrote extensive observation reports, drawing on field notes taken during the MDTM. In Hospital A, 16 online MDTMs were observed. During the observations in Hospital B, one or two researchers were physically present at one of its locations. At both locations, four MDTMs were observed, totaling eight MDTMs at Hospital B.

Towards the end of the observation period, semi‐structured interviews were conducted to explore participants' personal experiences with decision‐making during online MDTMs at Hospital A and hybrid MDTMs at Hospital B, respectively. Participants were asked about their perspectives on the meeting format, their experiences with decision‐making in online and hybrid settings (with particular attention to any changes they had observed since transitioning from an in‐person to an online format at Hospital A due to COVID‐19), and how they believed online and hybrid meetings could be further enhanced. Given the substantial workload at both hospitals, the decision was made not to conduct interviews with all MDTM participants. Instead, one or two medical specialists from each participating medical discipline were invited for an interview via email. In both hospitals, interviews were conducted with all chairs, along with 12 participants from Hospital A and 5 from Hospital B. Subsequently, data saturation was reached, as no new themes emerged. At the start of each interview, confidentiality was assured, and respondents provided consent. Interviews ranged from 25 to 60 min and were conducted in person, online, or by telephone, based on the interviewee's preference. All interviews were audio‐recorded and transcribed verbatim.

### 2.4 Ethics

The Medical Ethics Review Committee of one of the participating hospitals granted ethical approval for this study. Before observations began in both hospitals, participants were informed about the study's purpose and goals at the start of two MDTMs. This information was repeated at the start of each interview. Participants were further informed that their participation was voluntary, that confidentiality would be maintained, and that they could withdraw from the study at any time.

### 2.5 Data Analysis

Data analysis was conducted using the constant comparative method [18] and unfolded in two stages in MAXQDA. In the initial stage, a selection of interviews and observation reports was coded line by line, yielding an initial coding frame. This frame was then applied to subsequent transcripts and reports and further refined as new codes emerged. The second stage involved further analysis of this inductively coded dataset, focusing specifically on how the online and hybrid meeting setups affected participants' voice behavior. This process involved a further round of refinement and, ultimately, aggregation of codes.

The Consolidated Criteria for Reporting Qualitative Studies guidelines (COREQ) were followed in preparing the manuscript (see Supporting Material).

## 3 Results

Five major, overarching themes were found, which reflect the barriers participants experienced that hampered their ability and willingness to exercise voice. Excerpts of the observations are reported in the results section below, while Table 1 includes excerpts of the interview data that further illustrate our findings.

**Table 1:** Exemplary quotes from interviews.

Theme	Sub‐dimension	Quote
Benefits of online and hybrid MDTMs	Enhanced accessibility	“I also work in a different hospital. On Wednesdays, I always had to rush from that hospital to this hospital to attend the MDTM. Now I can just dial in. It is much more relaxed.” (Participant 4, Hospital A)
		“One major advantage is that you can still join the MDTM, even if you're not physically present at the hospital.” (Participant 6, Hospital A)
	Broader range of perspectives because of enhanced accessibility	“The major advantage is that more people can participate. This leads to new input, unique insights that we probably would not have otherwise.” (Participant 5, Hospital B)
		“Ultimately, you want enough people to join the MDTM because each person may have a unique perspective. New perspectives always benefit the decision‐making process.” (Chair 1, Hospital B)
**Experienced barriers to participants' voice behavior**		
Lack of non‐verbal communication	Cross‐talking	“During face‐to‐face meetings, you observe much more non‐verbal communication. When someone speaks, you can see what others think, and the transition between people is more natural. People just see: now this person is speaking, now that one will take the floor. It's less chaotic. In digital meetings, people often want to say things simultaneously, and then it goes like: ‘you go first’, ‘no, you go first!’ During in‐person meetings, this occurred less frequently. Then, people raised their hands and the chair would say: ‘first [name], then [name].” (Participant 10, Hospital A)
	Difficulties with signaling and gauging others' perspectives	“During in‐person meetings, you could observe the non‐verbal communication much more easily. You could tell when someone wanted to speak. Now, it's much more chaotic, as people often start talking simultaneously.” (Participant 1, Hospital A)
		“When people start talking simultaneously, it can be challenging to find a ‘spot’ to talk. I regularly think: ‘Well, whatever, forget it. I won't say anything anymore.’” (Participant 7, Hospital A)
		“During in‐person meetings, you could see someone's non‐verbal communication, so you at least had some understanding of whether they agreed or disagreed with something that was said. But that is no longer the case now that the meeting is online” (Chair 1, Hospital A)
		“We have people at two locations, and it is quite difficult to know what is happening at the other location. You can't see people's facial expressions, so you don't know how they react and if they would like to say anything.” (Chair 2, Hospital B)
	Difficulties with identifying potential allies	“Neurosurgeons sometimes say: ‘The patient is in good condition. We'll go for surgery.’ But that is not always a feasible option, in my opinion. In the past, my colleagues and I used to look at the pathologist who sat diagonally across from us. If he would make eye contact with us, we would know he doesn't agree with the surgeons either. I would then say something, and he would respond, and then a discussion would unfold. But now that the MDTM is online, it is difficult to have such quick exchanges, making it more difficult for us to speak up.” (Participant 12, Hospital A)
		“When the meeting was in‐person, you could see if someone agreed with something or not by looking at someone's facial expressions. So, you knew whether people would agree with you or not. And that made it easier to speak up.” (Participant 8, Hospital A)
Inadequate technology	Technical glitches	“Two weeks ago, we weren't able to share our screens. Only after 15 min, this problem was resolved, which meant that we had to discuss eight or nine patients in 30 min, instead of 45. (…) Such time pressure is never good because people are less likely to add nuances, offer different perspectives. At least, I notice that I am less likely to do so in case of time pressure.” (Chair 2, Hospital A)
		“The computers work suboptimal, causing delays and the MDTM running late. We have a long list of patients to be discussed so that is really undesirable.” (Participant 1, Hospital B)
	Minutes not visible	“Previously, there was a screen in the middle of the room displaying a patient's health record, so everyone could see what was being recorded. We miss that now. It's nice to be able to observe and check if what was said is written down correctly.” (Chair 2, Hospital A)
		“The minutes are currently not visible and that is unfortunate. Seeing the minutes allows you to unanimously reach a conclusion. If you see “operation” written down, you can immediately speak up in case you disagree.” (Participant 2, Hospital B)
Loss of focus		“It is quite easy to start doing other things, like checking your email, instead of paying attention to the discussion.” (Participant 8, Hospital A)
		Respondent: “People sit in the room but don't really pay attention.”
		Interviewer: “How do you know?”
		Respondent: “Well, last Monday [name neurosurgeon] was texting me during the MDTM.” (Participant 1, Hospital B)
Suboptimal layout of the meeting room		“Someone first has to activate the microphone button before you can speak. By the time that happens, the conversation has often already moved on. So, you lag behind all the time. That's frustrating. When it is really necessary, I make my voice heard. But sometimes, when it is a minor point or a subtle nuance, I remain silent.” (Participant 5, Hospital B)
		“You want everyone to be on board, but when the majority participates in person, you'll probably see that those who join remotely will be less involved in the discussions.” (Chair 3, Hospital A)
Difficulties with building relationships		“When you're new, it is quite inconvenient that you don't know these people and don't really have the chance to get to know them. I notice that I am a bit cautious in my communication and never really say: ‘I don't agree with this and this.” (Participant 9, Hospital A)

Participants in both hospitals believed online and hybrid MDTMs had several advantages compared to ‘traditional’ in‐person meetings. Participants from Hospital A perceived that meeting accessibility had improved since the MDTM switched to an online format, as it became easier to attend. Participants, for instance, reported that they could still participate in the MDTM even when they were not physically located on hospital premises. Participants from Hospital B added that the hybrid meeting format enabled a larger group of healthcare professionals to attend, fostering a diversity of perspectives that could enrich the discussions and decision‐making processes.

Given the aforementioned benefits, most participants believed online and hybrid meetings were here to stay. However, the results show that the online and hybrid meeting set‐up did have an impact on participants' ability and willingness to exercise voice.

### 3.1 Lack of Non‐Verbal Communication

Respondents from both hospitals experienced a lack of non‐verbal communication during online and hybrid meetings. This lack of nonverbal cues, such as body language, facial expressions, and eye contact, made it challenging for participants to make themselves heard. This manifested itself in three ways.

#### 3.1.1 Cross‐Talking

In both hospitals, attendees could not always see their fellow participants clearly on the screen. This was either due to limitations in the videoconferencing software, which allowed only five attendees to be visible on screen simultaneously (Hospital A), or to participants being seated far from the camera (Hospital B). Because participants could not see one another, including their facial expressions and body language, cross‐talking frequently occurred, with two or more attendees speaking simultaneously.

Observations revealed that, in some situations where cross‐talk occurred, the chairs intervened and, through turn‐taking, attempted to ensure that those who wished to speak could do so. However, because meeting attendees were not fully visible to the chairs, the latter could not always see which participants had begun to speak. As a result, individuals who wished to speak (and had tried to do so) were sometimes not invited by the chair to voice their perspectives, thereby preventing many of them from speaking. For instance, during an MDTM at Hospital A, multiple participants simultaneously voiced their perspectives when discussing a patient's diagnosis. To facilitate a structured discussion, the chair invited the two participants, who had appeared on screen when they began speaking, to present their viewpoints. After both did so, the chair proceeded with the discussion and invited other participants to share their perspectives on potential treatment options. While doing so, one of the oncologists spoke up and indicated that she had tried to speak simultaneously with the other two attendees, but that the chair had not yet asked for her input. The chair promptly apologized for unintentionally overlooking this participant. During the interviews, respondents reported similar experiences and explained that cross‐talk impeded their voice behavior in meetings.

#### 3.1.2 Difficulties With Signaling and Gauging Others' Perspectives

The experienced lack of nonverbal cues further made it difficult for participants to signal to the chair that they had something to say and for the chairs to identify who wished to contribute to the discussion. To elaborate, both participants and chairs reported during the interviews that nonverbal cues, such as making eye contact or subtly raising a hand, signaled participants' desire to speak. This signaling was considered crucial for maintaining a smooth and natural meeting flow. Chairs further described how nodding or raising an eyebrow served as indicators of agreement or disagreement, guiding their decisions about when to invite input. However, during online and hybrid meetings, these nonverbal cues would go largely unnoticed because not all participants were clearly visible on screen. Participants found it difficult to make initial contributions, particularly in ongoing discussions, while chairs had difficulty determining whether a participant wanted to speak.

#### 3.1.3 Difficulties With Identifying Potential Allies

Moreover, participants from Hospital A, particularly those lower in status and hierarchy, such as nurses and junior doctors, described how the lack of nonverbal cues hindered their ability to identify potential allies. During the interviews, these participants mentioned that they struggled to make their voices heard, as they had experienced on prior occasions that their input was not always being listened to or incorporated into the decision‐making process. Yet, their confidence to speak up increased when they believed they would receive support for their voice. Hence, nurses and junior doctors described how they searched for ‘potential allies’ when the meeting was still in person by ‘scanning’ the room and observing their colleagues' facial expressions and body language, or by exchanging glances with colleagues. However, with the transition to online meetings, identifying potential allies became more challenging, resulting in greater hesitancy among these attendees to voice their perspectives.

### 3.2 Inadequate Technology

The technology employed for the MDTMs further created hindrances for participants to express their perspectives, suggestions, and concerns. This was twofold.

#### 3.2.1 Technical Glitches

First, the observations indicated that technical glitches, including screen‐sharing malfunctions, audio issues, and microphone failures, frequently occurred. In such instances, two outcomes could occur. On the one hand, participants could opt to wait until the technical glitch had been resolved, resulting in a delayed start for the MDTMs. This meant that decisions had to be made within a more compressed timeframe, further increasing the already high workload and time pressure experienced by participants. Participants reported that, as a result, they were less inclined to speak up promptly and to introduce nuanced perspectives during the discussion, as they felt decisions had to be made quickly.

On the other hand, the meeting could commence, albeit with some discomfort and inconvenience. For example, in both hospitals, microphones malfunctioned, preventing some participants from sharing their insights and contributing to the discussion.

#### 3.2.2 Minutes Not Visible

The videoconferencing software allowed only one screen to be shared, and in both hospitals, it displayed only the MRI images. Input collected during the MDTM and recorded in patients' electronic health records was not visible to participants, which was considered a deficiency. Participants from both hospitals noted during the interviews that seeing this information on screen could facilitate decision‐making regarding patient cases, as it would help them voice their opinions and ideas when they disagreed with what was presented. Relatedly, participants believed that seeing this information on the screens could ensure that minor errors could be promptly rectified.

### 3.3 Loss of Focus

Participants further explained that they struggled to maintain focus during the MDTM. Comparing the online MDTM with in‐person meetings, one participant from Hospital A said, “It is easier to lose focus nowadays. You can now start checking your email or reviewing a patient file.” The observations painted a similar picture. Participants were observed glancing at another screen (Hospital A) or were seen typing WhatsApp messages on their mobile phones (Hospital B). Although this sometimes went unnoticed, for instance, when participants had turned off their cameras (Hospital A), in some cases, it came to light, as is illustrated in the example below.

Chair: “What are your thoughts about this, [name participant]?

‐‐‐ silence ‐‐‐‐

Chair: [name of participant]?

Participant: “Oh, sorry! I was just checking tomorrow's patient list. What was the diagnosis again?” (Observation, Hospital A).

As participants' attention waned, they failed to listen attentively to the information presented during the discussion, which, in turn, hampered their ability to constructively contribute to the ongoing discussion, for instance, by speaking up and sharing their views.

### 3.4 Suboptimal Layout of the Meeting Room

Interviews and observations at Hospital B revealed that participants who were not physically present in the same meeting room as the chairperson had difficulty making their voices heard because they had to rely on someone else to activate the audio. The sound was muted by default to prevent background noises from disrupting the meeting. However, the fact that participants couldn't immediately share their input, as they first had to ask the person operating the computer to activate the sound, meant that information was sometimes shared rather late—for instance, when the final decision had already been made or was about to be made. The delayed participation led participants, even those of higher status, such as neurosurgeons, to “choose their battles” when deciding whether to speak up; that is, they voiced their opinions only when they strongly disagreed with their colleagues at the other location.

Although this issue was not observed at Hospital A, participants nonetheless referred to the meeting setup. Specifically, participants expressed a strong preference for online MDTMs because they feared that, in hybrid meetings, those who are physically present might dominate the conversation, potentially reducing participation from remote attendees.

### 3.5 Difficulties With Building Relationships

Finally, participants from Hospital A, who had little work experience and were new to the MDTM, described that the online setting made it challenging to build relationships. Knowing other attendees was considered important as it helped participants feel comfortable, thereby enhancing their willingness to speak up. When the MDTM was still conducted in person, it was easier to interact with colleagues from other departments, as there was room for informal conversations before and after the MDTM. In the online environment, such informal moments did not occur, which participants believed hampered relationship development. As a result, participants, particularly those new to the MDTM, reported being cautious about voicing their perspectives.

## 4 Discussion

Prior studies have shown that MDTM participants may have difficulty exercising voice behavior during in‐person MDTMs [10, 11, 12]. The increasing digitalization and the COVID‐19 pandemic have led to an increase in online and hybrid meetings, introducing potential new obstacles to participants' voice behavior [9], which we aimed to explore in the present study. Drawing upon interviews and observations, we have shown how (1) the absence of non‐verbal cues, (2) technological deficiencies, (3) a lack of focus, (4) a suboptimal layout of the meeting room, and (5) challenges in building relationships with other attendees pose challenges to participants' voice behavior. Overall, our findings illustrate the multifaceted nature of obstacles to effective communication in MDTMs, with specific barriers inherent to online and hybrid meetings that are absent in in‐person settings.

A distinctive challenge in online and hybrid MDTMs, compared with in‐person meetings, is the absence of nonverbal cues [19]. Participants described how nonverbal cues, such as subtle gestures, a raised eyebrow, or eye contact, can influence meeting dynamics by revealing who is engaged, who seeks to contribute, and who may express agreement or dissent. This information is valuable, particularly for the chairperson, as it indicates who may wish to exercise voice. However, as shown in our results, during online and hybrid meetings, these cues may go largely unnoticed, which hampers the chair's ability to both invite input and structure discussions. From the participants' perspective, this demands proactive engagement, such as speaking up when the chair overlooks them and fails to invite their input. While we presented an example in our results of an attendee who did so, not every participant may feel at ease disrupting the meeting's natural flow and challenging the chair [9, 10].

Beyond its impact on meeting dynamics, our research further reveals that non‐verbal cues can be instrumental in empowering individuals, particularly those lower in hierarchy and status, to voice their perspectives. Specifically, the nurses and junior doctors from Hospital A described how finding ‘allies’ helped them speak up during MDTMs, as they believed these allies would support their voice messages. However, nonverbal cues went largely unnoticed online, thereby hindering lower‐status care professionals' ability to identify potential allies and, ultimately, hampering their voice behavior.

Respondents in both hospitals further described how technical issues and a lack of concentration contributed to the silence on their sides. Remarkably, these two obstacles have also been identified as hindering in‐person meetings. For instance, Oeppen et al. [20] discussed how the perception that a case discussion is taking too long can lead individuals to lose focus. Similarly, technical setbacks, such as the absence of essential MRI images, further impair the effectiveness of MDTMs [2, 7]. However, the transition to online and hybrid meeting formats has seemingly exacerbated these issues. For instance, online and hybrid meetings create unprecedented ease of distraction, with participants having their computers and phones readily available [21]. Likewise, the increased use of technology accentuates the looming threat of technological disruptions [14]. Thus, technical challenges and reduced focus remain prevalent and may hamper participants' voice behavior during in‐person, online, and hybrid MDTMs.

Finally, our study echoes scholarly work showing that building interprofessional relationships is more complex in online and hybrid settings [21], while studies suggest that such relationship‐building is crucial to effective voice behavior, particularly among those in lower‐status positions [11, 22]. In‐person meetings typically facilitate impromptu conversations, for instance, before or after the MDTM, thereby creating a sense of connection among care professionals [23]. Yet, the transition to an online format meant that such impromptu conversations occurred much less frequently, which was perceived as a significant deficiency of the online meeting format and an important barrier to voice behavior.

### 4.1 Practical Recommendations

In light of the study's results, several recommendations can be formulated to facilitate active participation and enable robust discussions during online and hybrid MDTMs. First, our study underscores the need to ensure that *all* participants can contribute equally to the discussions. The findings from Hospital B indicated that when a group of care professionals, including the chairperson, convenes in a single meeting room, there is a risk that the discussion will occur primarily among in‐person attendees, leaving participants joining online with little opportunity to speak up. Hence, it might benefit care teams to conduct MDTMs exclusively online. Related to the meeting set‐up, our study highlights the importance of a proficient videoconferencing software system that, ideally, shows (a) the meeting minutes, (b) imaging, and (c) all other attendees.

Our study further suggests that MDTMs benefit from a skilled chairperson who proactively engages attendees in discussions by asking for their input. Doing so could help participants feel included in the discussion and may help prevent disengagement, which is increasingly prevalent in online and hybrid meetings. Nonetheless, our study also reveals that chairpersons may encounter challenges in leading discussions. Further investigation could, therefore, examine the tactics chairpersons employ to elicit voice behavior and the prerequisites for effective implementation.

### 4.2 Strengths and Limitations

While combining observations and interviews enhanced the validity of our study and enabled us to assess whether our observations matched respondents' interpretations and experiences [14], there are some limitations to our research worth noting. First, we may not have been able to identify all the ways in which the online and hybrid meeting setup affects participants' voice behavior due to the limited number of MDTMs observed and interviews conducted. Relatedly, we have only studied neuro‐oncology MDTMs. To develop a better understanding of how the online and hybrid meeting setup affects participants' voice behavior, we recommend repeating this study in other hospitals and among patients with different pathologies.

## 5 Conclusion

In conclusion, voice behavior during online and hybrid MDTMs face challenges due to (1) the absence of non‐verbal cues, (2) technological deficiencies, (3) a lack of focus, (4) a suboptimal layout of the meeting room, and (5) difficulties in building relationships with other attendees. Overall, our findings underscore the need for tailored strategies to address the specific challenges posed by the digitalization of MDTMs.

## Conflicts of Interest

The authors declare no conflicts of interest.

## Supporting information


Supporting File

## Data Availability

The data that support the findings of this study are available on request from the corresponding author. The data are not publicly available due to privacy or ethical restrictions.
