# Supplementary material for: Barriers to Voice Behavior in Online and Hybrid Multidisciplinary Team Meetings: A Qualitative Study
Source: J Eval Clin Pract. 2026 Oct 11;32(7):e70614. doi: 10.1111/jep.70614 (PMC13654482; doi:10.1111/jep.70614)
Supplement: Supplementary file 1 [file JEP-32-0-s001.docx]

**Supplementary Material**

**Topic list interviews**

**Introduction**

- Brief explanation of the research objectives
- Mention that the interview is confidential and anonymous
- Obtain informed consent

**Personal background**

- Could you start by telling a bit about yourself? (Your role, years of work experience)

**Role in the MDTM**

- You participate as a neurosurgeon/neurologist/oncologist/radiotherapist/nurse/etc. in the MDTM. How would you describe your role within the team?
- How do you prepare for the MDTM?
- In your opinion, what is the goal (or goals) of an MDTM?
- When do you consider an MDTM to be successful?

**Voice**

- Do you make your voice heard during the MDTM when you have an idea, concern, or differing opinion?
  - If not, why?
  - What contributes to this?
- Do you feel encouraged to voice your opinions and share your input during the MDTM?
  - If yes, what contributes to this feeling?
  - If not, what should change to make you feel more comfortable speaking up?
- Does the set-up of the meeting (i.e., hybrid, online) affect your ability or willingness to make your voice heard?
  - If so, why?

*Specific questions for the chairs*

- As the chairperson, do you actively encourage other participants to voice their opinions during the MDTM?
  - How do you do this? Could you give an example?

**Questions about the chair**

- The MDTM is led by a chairperson. In your opinion, what are the characteristics of a good chairperson?
- Are there areas in which the current chairpersons could improve?

**Setting of the MDTM**

- How do you experience the online/hybrid setting of the MDTM?
  - What are benefits?
  - What are challenges?
- Does the online/hybrid setting affect the decision-making process during the MDTM? If so, how?
  - In the case of Hospital A: Is this different from decision-making during in-person MDTMs?

**Questions for the chairpersons**

- Why did you decide to become one of the chairs of the MDTM?
  - What do you enjoy about it?
- What distinguishes the role of the chairperson from that of a participant in the MDTM?
- Is it ever challenging to be the chairperson?
  - Can you provide an example?
- In your view, what are the characteristics of a good chairperson?
  - Reflecting on your own experiences, do you possess these characteristics?

**Closing**

- Do you think the decision-making process during the MDTM can be further improved? If so, how?
- Are there any other points that you would like to mention that we haven’t discussed?
- Thank you and closing.
